# DBG2OLC: Efficient Assembly of Large Genomes Using Long Erroneous Reads of the Third Generation Sequencing Technologies

**DOI:** 10.1038/srep31900

**Published:** 2016-08-30

**Authors:** Chengxi Ye, Christopher M. Hill, Shigang Wu, Jue Ruan, Zhanshan (Sam) Ma

**Affiliations:** 1 Department of Computer Science, Institute for Advanced Computer Studies, University of Maryland, College Park, MD 20742, USA; 2 Computational Biology and Medical Ecology Lab, State Key Laboratory of Genetic Resources and Evolution, Kunming Institute of Zoology, Chinese Academy of Sciences, Kunming, 650223 China; 3 Agricultural Genome Institute, Chinese Academy of Agricultural Sciences, No.7 Pengfei Road, Dapeng New District, Shenzhen, Guangdong 518120, China

## Abstract

The highly anticipated transition from next generation sequencing (NGS) to third generation sequencing (3GS) has been difficult primarily due to high error rates and excessive sequencing cost. The high error rates make the assembly of long erroneous reads of large genomes challenging because existing software solutions are often overwhelmed by error correction tasks. Here we report a hybrid assembly approach that simultaneously utilizes NGS and 3GS data to address both issues. We gain advantages from three general and basic design principles: (*i*) Compact representation of the long reads leads to efficient alignments. (*ii*) Base-level errors can be skipped; structural errors need to be detected and corrected. (*iii*) Structurally correct 3GS reads are assembled and polished. In our implementation, preassembled NGS contigs are used to derive the compact representation of the long reads, motivating an algorithmic conversion from a *de Bruijn* graph to an overlap graph, the two major assembly paradigms. Moreover, since NGS and 3GS data can compensate for each other, our hybrid assembly approach reduces both of their sequencing requirements. Experiments show that our software is able to assemble mammalian-sized genomes orders of magnitude more quickly than existing methods without consuming a lot of memory, while saving about half of the sequencing cost.

The Human Genome Project (HGP), which is perhaps the largest biomedical research project humans have ever undertaken, is responsible for greatly accelerating the advancement of DNA sequencing technologies^1^. Three generations of DNA sequencing technologies have been developed in the last three decades, and we are at the crossroads of the second and third generations of the sequencing technologies. Third generation sequencing (3GS) technology promises to significantly improve assembly quality and expand its applications in biomedical research and biotechnology development. However, lack of efficient and effective genome assembly algorithms has arguably been the biggest roadblock to the widespread adoption of 3GS technologies. 3GS long reads (averaging up to 5–20 kb per run at this time) usually have high error rates: ~15% with PacBio sequencing^2^, and as high as ~40% with Oxford Nanopore sequencing^3^. These high error rates make the assembly of 3GS sequences seem disproportionally complex and expensive compared to the assembly of NGS sequences. As a comparison, the whole genome assembly of a human genome using 3GS data was first reported to have taken half a million CPU hours^4^ compared to ~24 hours with Illumina NGS sequencing data^5^. Consequently, in practice, many applications of 3GS technology have been limited to re-sequencing bacteria and other small genomes^6^. While software for 3GS assembly has made important improvements^2^,^7^,^8^,^9^,^10^,^11^,^12^,^13^,^14^,^15^,^16^,^17^, especially for high coverage data, the software is still quite slow and not ideally suited for modest coverage data. Another major issue is that the sequencing cost of 3GS technology, while decreasing with time, is still at least an order of magnitude more expensive than the popular Illumina NGS sequencing at the time of this work.

While the evolution of genome assembly software solutions has been influenced by multiple factors, the most significant one has been the length of the sequences^18^. Although increasing sequence lengths may simplify the assembly graph^6^, the sequence length also has a critical impact on the computational complexities of genome assembly. Computational biologists have historically formulated the genome assembly problem as a graph traversal problem^18^,^19^,^20^, *i.e.,* searching for a most likely genome sequence from the *overlap graph* of the sequence reads in the case of the first generation sequencing technology. The *string graph* and the *best overlap graph* are specific forms of the *Overlap-Layout-Consensus* (OLC) paradigm that are more efficient by simplifying the global overlap graph^19^,^21^,^22^. The read-based algorithms, aiming to chain the sequencing reads in the most effective way, are computationally expensive because pair-wise alignment of the sequences is required to construct the overlap graph. This issue was tolerable for the relatively low amount of sequences produced from the low-throughput first generation sequencing technologies, but quickly became overwhelming with the enormous amount of short reads produced by high-throughput NGS data. The strategy of chopping the sequencing reads into shorter and overlapping *k*-grams (so-termed *k*-mers) and building links between the *k*-mers, was developed in the *de Bruijn* graph (DBG) framework to simplify NGS assembly. Assembly results are extracted from the linear (unbranched) regions of the *k*-mer graph in this approach^20^.

The overlap graph model or the OLC-based software packages, such as Celera Assembler^1^, AMOS^23^ and ARACHNE^24^, originally used for assembling the first generation sequence data, were also adopted for the NGS assembly before DBG-based approaches became the *de facto* standard. Newer 3GS technologies, including single-molecule, real-time sequencing (SMRT) and Oxford Nanopore sequencing, produce much longer reads than NGS. The longer reads from 3GS technology make the OLC approaches, which were originally used in the first generation genome assembly, feasible again. Nevertheless, the high error rates of current 3GS technologies render the existing OLC-based assemblers developed for relatively accurate sequences unusable. Similarly, the error-prone long reads make the DBG full of branches and therefore unsuitable for 3GS assembly. Faced with these challenges, the developers of 3GS technology have resorted to using error correction techniques^2^,^7^,^9^,^10^,^13^,^17^ to create high quality long reads and reusing the algorithms originally developed for the first generation sequence assembly. However, error correction for these long reads require extensive computational resources, even for small microbial genomes. Moreover, the high sequencing depth (usually 50x–100x) required by existing 3GS genome assemblers increases sequencing cost significantly, especially for large genomes. These issues have put 3GS technology at a severe disadvantage when competing against widely used NGS technology. In this article, we introduce algorithmic techniques that effectively resolve many of these issues. But first, we present a brief account of the existing genome assembly software technologies to put our contribution in proper context.

Researchers began with scaffolding approaches such as AHA^16^, PBJelly^15^ and SSPACE-LongRead^11^ to patch the gaps between high quality assembly regions, *i.e.*, first build a scaffold by aligning reads to the contigs and then use reads that span multiple contigs as links to build a scaffold graph. In ALLPATHS-LG^14^ and *Cerulean*^12^, long reads are used to find the best path in the *de Bruijn* graph that bridges the gaps between large contigs. Although these software packages have indeed achieved important advances for 3GS genome assembly, resolving intricate ambiguities is inherently difficult and can lead to structural errors. Furthermore, the underlying graph search algorithms usually have *exponential* complexity with respect to the search depth and thus, scales poorly; highly repeating regions (such as long repeats of simple sequences) will lead to large search depths and are not resolvable. In addition, the more powerful read overlap graph structure (of the long reads) was not fully explored in all these approaches. Often these algorithms rely on heuristics such as contig lengths and require iterations^12^,^14^. To circumvent these important issues associated with the hybrid approach, a Hierarchical Genome-Assembly Process (HGAP)^13^ was developed using a non-hybrid strategy to assemble PacBio SMRT sequencing data, which does not use the NGS short reads. HGAP contains a consensus algorithm that creates long and highly accurate overlapping sequences by correcting errors on the longest reads using shorter reads from the same library. This correction approach was proposed earlier in the hybrid setting and is widely used in assembly pipelines^2^,^9^,^10^,^17^. Nonetheless, this non-hybrid, hierarchical assembly approach requires relatively high sequencing coverage (50x–100x) and substantial error correction time to obtain satisfactory results. It is noteworthy that most of the algorithms we reviewed here were originally designed for bacterial-sized genomes. Though recent advancements in aligning erroneous long reads^6^,^25^ have also shortened the computational time of 3GS assembly, running these programs on large genomes, especially mammalian-sized genomes, usually imposes a large computational burden (sometimes up to 10^5^ or 10^6^ CPU hours) more suited to large computational clusters and well beyond the capability of a typical workstation.

In this study, we design algorithms to enable efficient assembly of large mammalian-sized genomes. We observe that per-base error correction of each long erroneous reads and their pair-wise alignment takes a significantly large portion of time in existing pipelines, but neither of these is necessary at an initial assembly stage. If all sequencing reads are structurally correct (non-chimeic), one can produce a structurally correct draft genome and improve the base-level accuracy in the final stage, as was originally done in the OLC approach. Taking advantage of this observation, we develop a base-level correction-free assembly pipeline by directly analyzing and exploiting overlap information in the long reads. Unlike previous approaches, we use the NGS assembly to lower the computational burden of aligning 3GS sequences rather than just polishing 3GS data. This allows us to take advantage of the cheap and easily accessible NGS reads, while avoiding the issues associated with existing hybrid approaches mentioned previously. Meanwhile, since NGS and 3GS are independent of each other, the sequencing gaps in one type of data may be covered by the data from the other. The utilization of NGS data also lowers the required sequencing depth of 3GS, and the net result is reduced sequencing cost. Hence, we get the best of both worlds of hybrid and non-hybrid assembly approaches. Specifically, we map the DBG contigs from NGS data to the 3GS long reads to create *anchors* for the long reads. Each long read is (lossily) compressed into a list of NGS *contig identifiers*. Because the compressed reads are often orders of magnitude shorter than the original reads, finding candidate overlaps between them becomes a simple bookkeeping problem and the approximate *alignments* and *overlaps* can be calculated cheaply with the help of the *contig indentifiers*. An overlap graph is constructed by chaining the best overlapped-reads in the compressed domain. The linear unbranched regions of the overlap graph are extracted and uncompressed to construct the draft assembly. Finally, we polish the draft assembly at the base-level with a consensus module to finish the assembly. Overall, compared with the existing approaches, our algorithm offers an efficient algorithmic solution for assembling large genomes with 3GS data in terms of computational resources (time and memory) and required sequencing coverage while also being robust to sequencing errors. Furthermore, our pipeline utilizes the reads overlap information directly and provides an efficient solution to the traditional read threading problem, which is valuable both theoretically and practically even for the NGS assembly^20^,^26^.

## Methods and Implementations

Our algorithm starts with linear unambiguous regions of a *de Bruijn* graph (DBG), and ends up with linear unambiguous regions in an overlap graph (used in the Overlap-Layout-Consensus framework). Due to this property, we dub our software DBG2OLC. The whole algorithm consists of the following five procedures, and we implement them as a pipeline in DBG2OLC. Each piece of the pipeline can be carried out efficiently.

**Figure 1:**
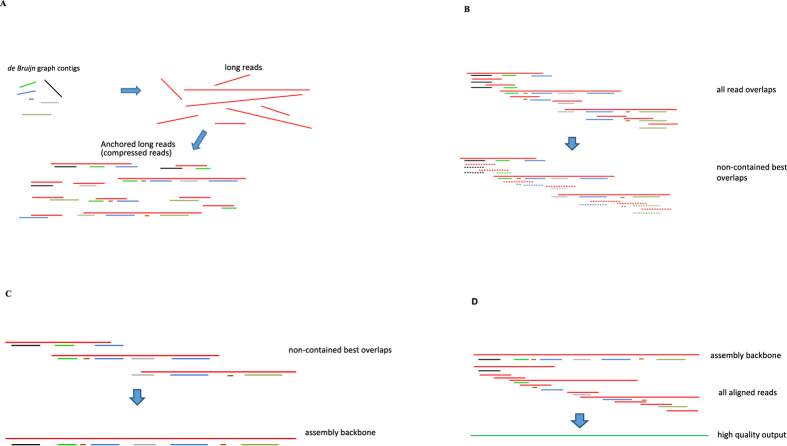
(**A**) Map *de Bruijn* graph contigs to the long reads. The long reads are in red, the *de Bruijn* graph contigs are in other colors. Each long read is converted into an ordered list of contigs, termed compressed reads. (**B**) Calculate overlaps between the compressed reads. The alignment is calculated using the anchors. Contained reads are removed and the reads are chained together in the best-overlap fashion. (**C**) Layout: construct the assembly backbone from the best overlaps. (**D**) Consensus: align all related reads to the backbone and calculate the most likely sequence as the consensus output.

**Figure 2:**
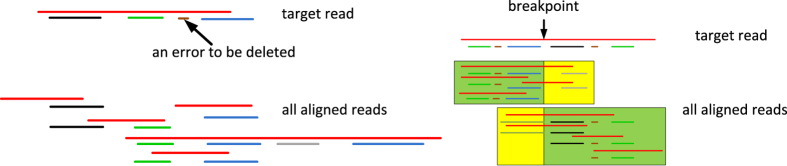
Reads correction by multiple sequence alignment. The left portion shows removing a false positive anchoring contig (brown) that appears only once in the multiple alignment. The right portion shows detection of a chimeric read by aligning it to multiple reads. A breakpoint is detected as all the reads can be aligned with the left portion of the target read are not consistent with all the reads that can be aligned with the right portion of the target read.

1. Construct a *de Bruijn* graph (DBG) and output contigs from highly accurate NGS short reads.
2. Map the contigs to each long read to anchor the long reads. The long reads are compressed into a list of *contig identifiers* to reduce the cost of processing the long reads (Fig. 1A).
3. Use multiple sequence alignment to clean the compressed reads, and remove reads with structural errors (or so-called chimeras) (Fig. 2).
4. Construct a best overlap graph using the cleaned compressed long reads (Fig. 1B).
5. Uncompress and chain together the long reads (Fig. 1C), and resort to a consensus algorithm to convert them into DNA sequences (Fig. 1D).

Details for procedure (2–5) are explained below. The explanation of procedure (1) can be found in our previous *SparseAssembler* for NGS technology^5^ and is omitted here.

## Availability

The source code and a compiled version of DBG2OLC is available in the following site: https://github.com/yechengxi/DBG2OLC.

## Reads Compression

We use a simple *k-*mer index technique to index each DBG contig and map the pre-assembled NGS contigs back to the raw sequencing reads as anchors. The *k*-mers that appear in multiple contigs are excluded in our analysis to avoid ambiguity. Empirically for PacBio reads, we found that using k = 17 were adequate for all our experiments. For each 3GS long read, we report the matching contig identifiers as an ordered list. A contig identifier is reported if the number of uniquely matching *k*-mers in that contig is above a threshold, which is adaptively determined based on the contig length. We set this threshold in the range of (0.001~0.02)*Contig_Length. This easily tuneable threshold parameter allows the user to find a balance between sensitivity and specificity. With low coverage datasets, this parameter is set lower to achieve better sensitivity; otherwise it is set to higher to enforce better accuracy. In all our experiments, the contigs are generated with our previous *SparseAssembler*^5^.

After this procedure, each read is converted into an ordered list of *contig identifiers*. An example of such a list is {Contig_a, Contig_b}, where Contig_a and Contig_b are identifiers of two different contigs. We also record the orientations of these contigs in the mapping. This compact representation is a lossy compression of the original long reads. We term the converted reads as compressed reads in this work. A compressed read is considered to be equivalent to its reverse complement, and the same compressed reads are then collapsed. Since a *de Bruijn* graph can efficiently partition the genome into chunks of bases as contigs, this lossy compression leads to orders of magnitude reduction in data size. Moreover, the compact representation can span through small regions with low or even no NGS coverage; these important *gap regions* in NGS assembly can be covered by 3GS data. Likewise, small 3GS sequencing gaps may be covered by NGS contigs. These sequencing gaps will be bridged in the final stage. Similarity detection between these compressed reads becomes a simpler bookkeeping problem with the identifiers and can be done quickly with low memory. To demonstrate the effectiveness of this strategy, we ran it over five datasets including genomes of different sizes and different sequencing technologies (Table 1, resources can be found in the Supplementary Materials). The compression usually leads to three factors of reduction in read length with 3GS.

## Ultra-fast Pair-wise Alignments

Most existing algorithms rely on sensitive algorithms^27^,^28^ to align reads to other reads or assemblies. In our approach, since the compressed reads are usually much shorter than the original reads, alignments of these compressed reads can be calculated far more efficiently. We adopt a simple bookkeeping strategy and use the contig identifiers to build an inverted-index. Each identifier points to a set of compressed reads that contain this identifier. This inverted-index helps us to quickly select the potentially overlapping reads based on shared contig identifiers. Alignments are calculated only with these candidate compressed reads. The alignment score is calculated using the Smith-Waterman algorithm^29^; the contig identifiers that can be matched are positively scored while the mismatched contig identifiers are penalized. Scores for match/mismatch are calculated based on the involved contig lengths or the number of matching *k*-mers in the previous step. With the compressed reads, our algorithm can finish pair-wise alignments in a small amount of time.

As discussed previously, state-of-the-art assembly pipelines usually resort to costly base-level error correction algorithms to correct each individual read^2^,^7^,^8^,^10^,^13^, which they then feed into an existing assembler. However, an *important finding* of this work is that per-base accuracy may not be a major roadblock for assembly contiguity. Rather, the *chimeras* or *structural sequencing errors* are the major “hot spots” worth putting major effort into. Without cleaning these chimeras, the overlap relations include many falsely generated reads and will lead to a tangled overlap graph. To resolve this issue, we compute multiple sequence alignments (MSA) by aligning each compressed read with all other candidate compressed reads. With MSA we can detect the chimeric reads and the spurious contig identifiers in each read (Fig. 2). Both of these errors are cleaned up. The major side effect of this correction is a slightly increased requirement of the 3GS data coverage so that each compressed read can be confirmed by at least another one. The remaining minor errors (mostly false negatives) in the cleaned compressed reads will be tolerated by the alignment algorithm. In our experiments, we noticed that this algorithm is accurate enough to find high quality overlaps and can be used for constructing draft genomes as assembly backbones.

## Read Overlap Graph

Compared with most hybrid approaches that used long reads to link together the short read contigs, our approach takes the unorthodox way–we use the short read contigs to help link together the long reads. We construct a best overlap graph^21^ using the above-described alignment algorithm with the compressed reads. In the best overlap graph, each node represents a compressed read. For each node, the best overlapped nodes (one before and the other after) are found based on the overlap score, and the links between these nodes are recorded. The overlap graph is calculated in two rounds (Fig. 1B). In round *1*, all the contained nodes (with respect to other nodes) are filtered off. For example, {Contig_a, Contig_b} is removed if {Contig_a, Contig_b, Contig_c} is present. With this strategy, alignments with repeating and contained nodes are avoided. In round *2*, all suffix-prefix overlaps among the remaining nodes are detected with the alignment algorithm. Nodes are chained one to another in both directions and in the best overlapped fashion. Graph simplification is applied to remove tiny tips and merge bubbles in the best overlap graph. Truly unresolvable repeats result in branches in the graph^21^ and will be kept as the assembly breakpoints.

Note that constructing the overlap graph with the compressed reads offers us several major benefits. (1) Long read information is sufficiently utilized. (2) The costly long read alignments are accelerated with the easily available NGS contigs. (3) The expensive graph search algorithms (with exponential complexity to the search depth) often used for graph resolving in many existing genome assembly programs are no long needed in our software.

## Consensus

It is noteworthy that only in this final stage that the compressed reads are converted back to the raw nucleotide reads for polishing purpose. Linear unbranched regions of the best overlap graph encode the unambiguously assembled sequences. Uncompressed long reads that lie in these regions are laid out in the best-overlapped fashion and patched one after another (Fig. 1C). NGS contigs are included when there is a gap in the 3GS data. Reads that are related to each backbone are collected based on the contig identifiers. A consensus module is finally called to align these reads to each backbone and calculate the polished assembly (Fig. 1D). To polish the 3GS assembly backbone, we use an efficient consensus module *Sparc*^30^*. Sparc* builds an efficient sparse *k*-mer graph structure^5^ using a collection of sequences from the same genomic region. The heaviest path approximates the most likely genome sequence (consensus) and is found in a sparsity-induced reweighted graph.

## Results

We conducted a comprehensive comparison on a small yeast genome (12 Mbp) dataset to provide a scope of the performance of each software program we compared in this study. Since most other programs do not scale linearly with the data scale and require thousands of hours per-run on genomes larger than 100 Mbp, the readers are encouraged to read through their original publications for the performance results of those programs.

As a side note, the advent of 3GS long reads has raised the bar to a higher level compared with previous sequencing techniques: existing reference genomes usually contain a large number of structural errors and/or variations that can surpass the number of assembly errors using the long reads. In most cases we select assemblies by other assemblers with more coverage (~100x) as references. If high quality reference genome is available, thorough evaluations of our algorithm show that DBG2OLC can provide high quality results with fewer structural errors and comparable per-base accuracy. This has been recently demonstrated in the case study of *D. melanogaster* genome by Chakraborty *et al.*^31^ who compared our pre-released software with other premier programs for 3GS data. In this paper, we demonstrate results on some other well-studied species and use existing high coverage assemblies as quality checks. On medium to large genomes, DBG2OLC can produce comparably good results with one to two orders of magnitude less time and memory usages than most existing pipelines. A draft assembly (without polishing) of a 3 Gbp *H. sapiens* can be finished in 3 CPU days with our pipeline, utilizing 30 × 3GS and 50x NGS data. This computational time is roughly comparable to many existing NGS assemblers. The time consumption of each step running different genomes can be found in Table 2.

We compared our algorithm results with Celera Assembler (CA, version 8.3rc2), PacBioToCA (in CA8.3rc2)^2^, ECTools^9^, MHAP (in CA8.3rc2)^7^, HGAP (in SMRT Analysis v2.3.0)^13^ and Falcon assembler (v0.3.0), which are well recognized as the best-performed genome assemblers for 3GS technologies. Data from PacBio SMRT RS-II (the currently leading platform of 3GS technology) was used to perform the comparative experiments (50x Illumina MiSeq reads were additionally used for PacBioToCA and DBG2OLC, the two hybrid methods). The experiments are run on a server with eight Intel Xeon E7-8857 v2 CPUs (each has 12 cores) and 2 TB memory. For all DBG2OLC experiments in this paper we used SparseAssembler (Ye *et al.*^5^) to preassemble 50x Illumina short reads into contigs and then to compress the 3GS reads. Similarly, Celera Assembler was used to assemble the same short reads into contigs for ECTools. Unassembled short reads were fed into PacBioToCA according to its specification. At the time of this work, 50x Illumina reads cost less, and also can be obtained more easily, than 1 × 3GS reads. Celera Assembler could be run with uncorrected reads on small datasets, so we run it as a baseline.

It is noteworthy that in our current implementation, most of the computation time (~90%) is spent on the consensus step, in which BLASR^28^ is called to align all raw reads to the assembly backbone. Since the alignments are multi-threaded, the wall time can be reduced depending on the available threads. The consensus step is relatively independent in genome assembly and is open to any future improvements and accelerations. The overall computational time of the whole pipeline scales near linearly to the data size, which is a highly valuable property to large-scale genome assembly problems. Using 10x–20x coverage of PacBio sequence data, we obtained assembly N50s that are significantly (>10x) better than Illumina data alone (Table 1). The datasets, commands, and parameters can be found in the Supplementary Materials. We used QUAST 3.0^32^ in its default setting to evaluate the assembly results; these are reported as the NGA50, per-base identity rates and misassembly errors. In analyzing 3GS assembly results, the NGA50 is a measure of the average length of high quality region before reaching a poor quality region in the assembly. The identity rates were calculated by summarizing the single base mismatches and insertion/deletion mismatches. Relocations, inversions, and translocations are regarded as misassembly errors^32^. The alignment dot plots can be found in the Supplementary Materials. The nearly perfect diagonal dot plots indicate that DBG2OLC can produce structurally correct assemblies from as low as 10x long read data.

For the yeast dataset we picked an assembly from 454 data (NCBI Accession No.: GCA_000292815.1) and another assembly generated using MHAP with high coverage data as references. DBG2OLC takes advantage of different sequencing types and obtains the most contiguous results using 10x–40x data with comparable levels of accuracy (Table 3). Some non-hybrid assemblers are not able to fully assemble the yeast genome with 10x–20x PacBio data. It is also worth mentioning a caveat in many current hybrid error correction approaches. These pipelines use NGS contigs to correct the 3GS reads, which seem to have improved the accuracy of each individual 3GS read. However, the errors in NGS contigs may have corrupted the originally correct 3GS reads and lead to consensus errors in the final assembly. For example, we notice the identity rates of the ECTools assembly are higher when aligned to the 454 reference, contrary to all other pipelines. With high enough coverage (and significantly increased sequencing cost), the 3GS self-correction based assembly methods produce better assembly results. Since our pipeline has a major advantage in low coverage data and efficiency, it is expected to scale well to large genomes where low coverage data and computational time becomes major concerns.

We tested DBG2OLC on other medium to large genomes from PacBio sequencers (Table 4). On the *A. thaliana* genome (120 Mbp), the computations with DBG2OLC finish in one hour, with an additional hour spent constructing the initial NGS contigs. The consensus module takes another 10–20 CPU hours to get the final assembly. The peak memory usage is 6 GB. In comparison, existing pipelines can take over one thousand CPU hours with problems of this scale. On a large 54x human (*H. sapiens*) dataset, DBG2OLC is able to produce an assembly with high contiguity starting from 10x PacBio data (NG50 433 kbp) and DBG contigs generated from 50x Illumina reads (Table S1 in Supplementary Materials). To produce a better assembly, the longest 30x of the reads in this dataset (mean length 14.5 kbp) are selected (Table S2 in Supplementary Materials). DBG2OLC occupies 70 GB memory to store the 17-mer index, and takes 37 CPU hours to compress and align the 30x longest PacBio reads. The pair-wise alignment takes only 3 hours and takes less than 6 hours on the full 54x dataset. The final consensus takes roughly 2000 CPU hours. In an initial report by PacBio scientists, the overlapping process took 405,000 CPU hours^4^. Our final assembly quality (N50 = 6 Mbp) is comparable to the state-of-the-art results obtained using orders of magnitude more resources. When evaluating this assembly, QUAST 3.0 can take weeks to finish the full evaluation even on our best workstation. We therefore only align our assembly to the longest 500 Mbp assembly generated by the Pacific Biosciences, and report the NGA50 and identity rate in this portion.

DBG2OLC was also tested on an Oxford Nanopore MinION sequencing dataset (Table 4). According to initial studies, this type of data has higher (up to ~40%) error rates^3^ compared to PacBio SMRT sequencing. However, we find DBG2OLC still successfully assembled the *E. coli* into one single contig. The polished assembly has an error rate of 0.23%. The dot plot of the alignment of the assembly to the reference can be found in Fig. 13 of the Supplementary Materials.

Compared with the state-of-the-art assemblers for 3GS technologies, our proposed method produces assemblies with high contiguity using lower sequencing coverage and memory, and is orders of magnitude faster on large genomes. Its combination of different data types leads to both computation and cost efficiency. These advantages are gained from three general and basic design principles: (*i*) Compact representation of the long reads leads to efficient alignments. (*ii*) Base-level errors can be skipped, but structural errors need to be detected and cleaned. (*iii*) Structurally correct 3GS reads are assembled and polished. DBG2OLC is a specific and simple realization of these principles. Interestingly, this implementation builds a nice connection between the two major assembly frameworks, and even though DBG2OLC is majorly developed for 3GS data, this strategy of compression and converting a *de Bruijn* graph to an overlap graph is general and can be used for popular NGS data. A preliminary showcase on a purely NGS dataset can be found in the Supplementary Materials. The strategy of compressing long reads and performing the most computationally expensive tasks in the compressed domain strikes a balance between the DBG and OLC frameworks.

## Summary and Discussion

In summary, we have built and validated a new *de novo* assembly pipeline that significantly reduces the computational and sequencing requirements of 3GS assembly. We demonstrate that the erroneous long reads can be directly assembled and can lead to significantly improved assembly without base-level error correction. This strategy, first publicly demonstrated in our pre-released pipeline in 2014, has paved the road for several subsequent development attempts on efficient utilization of 3GS data and promises even more efficient 3GS assemblers. Another major finding in developing DBG2OLC is that 3GS technologies generate chimeric reads, and the problem seems to be severer with the PacBio platform. These structural errors lead to tangles in the assembly graph and greatly hamper the assembly contiguity. The most straightforward way to clean up the chimeric reads resorts to multiple sequence alignment, as implemented in DBG2OLC, which leads to a slightly increased coverage requirement. This limitation will serve as the starting point for future development. We conjecture that near perfect assemblies can be reached with even lower coverage if the chimeras/structural errors can be removed.

## Additional Information

**How to cite this article**: Ye, C. *et al.* DBG2OLC: Efficient Assembly of Large Genomes Using Long Erroneous Reads of the Third Generation Sequencing Technologies. *Sci. Rep.* **6**, 31900; doi: 10.1038/srep31900 (2016).

## Supplementary Material

Supplementary Information

## Figures and Tables

**Table 1:** The demonstration of the compression ratio on various datasets.

Datasets	Sequencing Technology	Average Raw Read Length	NGS Contig N50 (DBG *k*-mer size)	Average Compressed Read Length	Compression Ratio
*S. cer w303*	PacBio	4,734	31,233 (*k* = 51)	7	1:676
*A. thaliana ler-0*	PacBio	5,614	2,264 (*k* = 51)	8	1:702
*H. sapiens*	PacBio	14,519	3,115 (*k* = 51)	11	1:1320
*E.coli K12*	Oxford Nanopore	6,597	3,303 (*k* = 21)	4	1:1649
*E.coli K12*	Illumina Miseq	150	3,303 (*k* = 21)	2	1:75

**Table 2:** Computation time of each procedure.

Species	Long Read Source	Short Read Assembly (CPU hr)	Compression (CPU hr)	Graph Construction (CPU hr)	Consensus (CPU hr)
*S. cer w303*	20x PacBio	0.1	0.03	0.005	2
*A. thaliana ler-0*	40x PacBio	1	0.6	0.2	18
*H. sapians*	30x PacBio	25	37	3	1600
*E. coli K12*	30x Nanopore	0.1	0.02	0.002	2

**Table 3:** Assembly performance comparison on the *S. cerevisiae* genome (genome size: 12 M bp).

Cov	Assembler	Time (h)	NG50	Contigs	NGA50 (454)	Identity (454)	Misass-emblies (454)	NGA50 (PacBio)	Identity (PacBio)	Misass-emblies (PacBio)	Longest	Sum
10x	MHAP*	—	—	—	—	—	—	—	—	—	—	—
	HGAP*	36.3	—	554	—	99.68%	105	—	99.77%	6	36,942	1,512,911
	CA*	15.1	85,728	289	68,030	97.49%	134	81,451	97.46%	13	448,177	12,285,888
	PacBioToCA	173.5	19,694	898	19,378	99.88%	112	18,689	99.90%	6	221,736	10,741,663
	ECTools	24.5	120,126	169	98,965	99.76%	324	109,640	99.73%	29	525,820	11,785,741
	Falcon*	1.3	—	675	—	99.23%	116	—	99.28%	4	36,616	4,137,485
	DBG2OLC	1.7	475,890	67	168,612	99.70%	408	355,269	99.81%	46	1,174,277	11,899,604
20x	MHAP*	17.1	241,394	87	155,221	99.70%	508	241,260	99.75%	22	490,764	12,123,145
	HGAP*	31.1	8,578	1,210	6,908	99.85%	307	7,619	99.90%	20	86,998	8,624,090
	CA*	42.4	371,115	165	201,649	98.83%	284	329,930	98.82%	21	680,599	13,052,212
	PacBioToCA	400.9	66,974	395	65,171	99.87%	157	65,171	99.91%	7	628,280	11,487,222
	ECTools	34.2	176,663	172	109,931	99.77%	565	150,351	99.74%	46	624,112	12,887,799
	Falcon*	3.5	110,083	180	93,385	99.38%	345	110,438	99.42%	15	281,041	10,583,868
	DBG2OLC	2.6	597,541	47	172,455	99.71%	440	576,287	99.88%	37	1,085,773	12,476,994
40x	MHAP*	36.6	614,363	65	243,012	99.91%	598	589,044	99.94%	24	1,090,578	12,356,826
	HGAP*	36.2	211,631	93	198,387	99.94%	528	348,754	99.99%	30	796,762	12,387,287
	CA*	115.2	365,912	114	160,867	99.66%	358	377,360	99.60%	11	769,189	15,171,228
	PacBioToCA	621.7	96,817	371	96,476	99.87%	178	94,480	99.91%	6	742,046	11,700,172
	ECTools	55.8	255,956	271	166,945	99.79%	891	214,377	99.76%	64	714,196	14,481,947
	Falcon*	11.2	614,509	58	247,745	99.72%	336	555,886	99.74%	10	1,069,920	12,116,235
	DBG2OLC	4.2	672,955	28	238,683	99.87%	431	544,679	99.90%	36	1,086,380	12,149,997
80x	MHAP*	13.5	751,122	43	248,079	99.91%	526	745,563	99.95%	10	1,537,433	12,350,704
	HGAP*	46.5	818,775	33	248,655	99.95%	534	678,552	99.99%	23	1,545,906	12,621,393
	CA*	236.0	430,552	75	201,397	99.80%	319	397,774	99.74%	12	984,295	16,571,250
	PacBioToCA	274.3	64,967	364	63,651	99.88%	45	62,268	99.91%	10	233,799	11,651,218
	ECTools	100.9	247,871	382	154,348	99.79%	1,470	164,839	99.76%	101	881,635	15,925,328
	Falcon*	34.7	810,136	99	247,480	99.81%	437	810,134	99.82%	24	1,537,463	12,681,860
	DBG2OLC	8.1	678,365	29	204065	99.92%	426	574,476	99.95%	35	1,089,897	12,209,592

^*^Assemblers that use only 3GS data.

**Table 4:** DBG2OLC assembly performance comparison on various genomes.

Genome	Size	Coverage	NG50	Contigs	NGA50	Identity	Misassemblies	Longest	Sum
*A. thaliana*	120 Mbp	10x PacBio	405,464	881	258,924	99.77%	704	1,549,329	119 Mb
		20x PacBio	2,431,755	306	926,138	99.90%	117	6,015,430	120 Mb
		40x PacBio	3,601,597	243	1,605,981	99.93%	131	15,473,059	129 Mb
*H. sapiens*	3.0 Gbp	10x PacBio	432,739	16,689	347,104	99.56%	—	3,507,306	2.97 G
		20x PacBio	1,886,756	9,757	1,416,766	99.82%	—	14,597,500	3.13 Gb
		30x longest PacBio	6,085,133	13,095	4,124,714	99.85%	—	23,825,526	3.21 Gb
*E. coli*	4.6 Mbp	30x Nanopore	4,680,635	1	1,850,974	99.77%	1	4,680,635	4.7 Mb
