# Supplementary material for: DBG2OLC: Efficient Assembly of Large Genomes Using Long Erroneous Reads of the Third Generation Sequencing Technologies
Source: Sci Rep. 2016 Aug 30;6:31900. doi: 10.1038/srep31900 (PMC5004134; doi:10.1038/srep31900)
Supplement: Supplementary Information [file srep31900-s1.pdf]

DBG2OLC: Efficient Assembly of Large Genomes Using Long Erroneous Reads of the Third Generation Sequencing Technologies

Chengxi Ye[1§], Christopher M. Hill[1], Shigang Wu[2], Jue Ruan[2], Zhanshan (Sam) Ma[3§]

[1]Department of Computer Science, Institute for Advanced Computer Studies, University of Maryland, College Park, MD 20742, USA.

[2]Agricultural Genome Institute, Chinese Academy of Agricultural Sciences, No.7 Pengfei Road, Dapeng New District, Shenzhen, Guangdong 518120, China.

[3]Computational Biology and Medical Ecology Lab, State Key Laboratory of Genetic Resources and Evolution, Kunming Institute of Zoology, Chinese Academy of Sciences, Kunming, 650223 China.

Correspondence email addresses: Chengxi Ye: cxy@umd.edu, Sam Ma: samma@uidaho.edu

Supplementary Materials

1. Source Code

The source code of SparseAssembler, DBG2OLC and Sparc can be found here:
https://github.com/yechengxi/SparseAssembler
https://github.com/yechengxi/DBG2OLC
https://github.com/yechengxi/Sparc
To compile, download the code into separate folders and use:

```
g++ -O3 -o SparseAssebmler *.cpp
g++ -O3 -o DBG2OLC *.cpp
g++ -O3 -o Sparc *.cpp
```

2. Datasets used in the paper

Table S1. Illumina datasets used in the paper

Datasets	Sequencing Type	Coverage Used	Illumina Data Source
S. cer w303	MiSeq	50x	http://schatzlab.cshl.edu/data/ectools/
A. thaliana ler-0	MiSeq	50x	http://schatzlab.cshl.edu/data/ectools/
H. sapiens	HiSeq	50x	Accession No.: SRR1283824
E.coli K12	MiSeq	50x	Accession No.: SRR826442, SRR826444, SRR826446, SRR826450

Table S2. PacBio/Nanopore datasets used in the paper

Datasets	PacBio Data Source
S. cer w303	http://schatzlab.cshl.edu/data/ectools/
A. thaliana ler-0	http://schatzlab.cshl.edu/data/ectools/
H. sapiens	http://datasets.pacb.com/2014/Human54x/fast.html
E. coli K12	http://gigadb.org/dataset/100102

Table S3. Reference genomes used in the paper

Datasets	Reference Data Source
S. cer w303	1. http://www.cbcb.umd.edu/software/PBcR/mhap/asm/yeast.quiver.all.fasta<br>2. Accession No.: GCA_000292815.1<br>ftp://ftp.ncbi.nlm.nih.gov/genomes/all/GCA_000292815.1_ASM29281v1/GCA_000292815.1_ASM29281v1_genomic.fna.gz
A. thaliana ler-0	http://www.cbcb.umd.edu/software/PBcR/mhap/asm/athal.quiver.all.fasta
H. sapiens	Accession No.: GCA_000772585.3<br>ftp://ftp.ncbi.nlm.nih.gov/genomes/all/GCA_000772585.3_ASM77258v3/GCA_000772585.3_ASM77258v3_genomic.fna.gz
E. coli K12	Accession No.: NC_000913<br>ftp://ftp.ncbi.nlm.nih.gov/genomes/all/GCA_000005845.2_ASM584v2/GCA_000005845.2_ASM584v2_genomic.fna.gz

3. Exemplary Assembly Commands

Step0. [Optional] Preparations:

We have provided code to help you select a subset of the reads:

https://github.com/yechengxi/AssemblyUtility

The utility functions can be compiled in the same way as the main programs. After compilation, you can use the following command to select a subset of reads from fasta/fastq files. Note that longest 0 is used here, if you set it to 1 it will select the longest reads.

./SelectLongestReads sum 600000000 longest 0 o Illumina_50x.fastq f Illumina_500bp_2x300_R1.fastq

./SelectLongestReads sum 260000000 longest 0 o Pacbio_20x.fasta f Pacbio.fasta

And you can use the following command to evaluate an assembly.

./AssemblyStatistics contigs YourAssembly.fasta

The program will generate two txt files containing essential statistics about your assembly.

Step1. Use a DBG-assembler to construct short but accurate contigs. Please make sure they are the **raw** DBG contigs **without** using repeat resolving techniques such as gap closing or scaffolding. Otherwise you may have poor final results due to the errors introduced by the heuristics used in short read assembly pipelines.

SparseAssembler command format:

./SparseAssembler GS [GENOME_SIZE] NodeCovTh [FALSE_KMER_THRESHOLD] EdgeCovTh [FALSE_EDGE_THRESHOLD] k [KMER_SIZE] g [SKIP_SIZE] f [YOUR_FASTA_OR_FASTQ_FILE1] f [YOUR_FASTA_OR_FASTQ_FILE2] f [YOUR_FASTA_OR_FASTQ_FILE3_ETC]

A complete example on the *S.cer* w303 dataset:

Download the Illumina reads from

ftp://qb.cshl.edu/schatz/ectools/w303/Illumina_500bp_2x300_R1.fastq.gz

Normally with ~50x coverage, NodeCovTh 1 EdgeCovTh 0 can produce good results.

./SparseAssembler LD 0 k 51 g 15 NodeCovTh 1 EdgeCovTh 0 GS 12000000 f ../Illumina_data/Illumina_50x.fastq

In this test run, the N50 is 29 kbp. As we have selected the beginning part of the sequencing file, which usually is of lower quality, the next step may help to improve the assembly quality.

[Miscellaneous]

For other more complex genomes or a different coverage, the first run may not generate the best result. The previous computations can be loaded and two parameters can be fine-tuned to construct a cleaner de Bruijn/ k-mer graph:

./SparseAssembler ***LD 1 NodeCovTh 2 EdgeCovTh 1*** *k 51 g 15 GS 12000000 f ../Illumina_data/Illumina_50x.fastq*

The N50 is improved to 32kbp in my run.

The output Contigs.txt will be used by DBG2OLC.

Step2. Overlap and layout. Feed DBG2OLC with the contig file in fasta format from the previous step (Contigs.txt in this example).

Download the PacBio reads from:

ftp://qb.cshl.edu/schatz/ectools/w303/Pacbio.fasta.gz

The basic command format of DBG2OLC is:

./DBG2OLC k [KmerSize] AdaptiveTh [THRESH_VALUE1] KmerCovTh [THRESH_VALUE2] MinOverlap [THRESH_VALUE3] Contigs [NGS_CONTIG_FILE] f [LONG_READS.FASTA] RemoveChimera 1

In the following example, the first 20x PacBio reads are extracted from the abovementioned file and we can assemble with:

./DBG2OLC k 17 AdaptiveTh 0.0001 KmerCovTh 2 MinOverlap 20 RemoveChimera 1 Contigs Contigs.txt f ../Pacbio_data/Pacbio _20x.fasta

In our test run, the N50 is 583kbp.

There are three major parameters that affect the assembly quality:

M = matched k-mers between a contig and a long read.

AdaptiveTh: adaptive *k*-mer matching threshold. If M < AdaptiveTh* Contig_Length, this contig cannot be used as an anchor to the long read.

KmerCovTh: fixed k-mer matching threshold. If M < KmerCovTh, this contig cannot be used as an anchor to the long read.

MinOverlap: minimum overlap score between a pair of long reads.

For each pair of long reads, an overlap score is calculated by aligning the compressed reads and score with the matching *k*-mers.

[Miscellaneous]

At this point, the parameters may be fine-tuned to get better performance. As with SparseAssembler, LD 1 can be used to load the compressed reads/anchored reads.

Suggested tuning range is provided here:

For 10x/20x PacBio data: KmerCovTh 2-5, MinOverlap 10-30, AdaptiveTh 0.001~0.01.

For 50x-100x PacBio data: KmerCovTh 2-10, MinOverlap 50-150, AdaptiveTh 0.01-0.02.

Some other less flexible or less important parameters:

k: *k*-mer size, 17 works well.

Contigs: the fasta contigs file from existing assembly.

MinLen: minimum read length.

RemoveChimera: remove chimeric reads in the dataset, suggest 1 if you have >10x coverage.

For high coverage data (100x), there are two other parameters:

ChimeraTh: default: 1, set to 2 if coverage is ~100x.

ContigTh: default: 1, set to 2 if coverage is ~100x.

These two are used in multiple alignment to remove problematic reads and false contig anchors. When we have high coverage, some more stringent conditions shall be applied as with the suggested parameters.

Step 3. Call consensus. Install *Blasr* and the consensus module (*Sparc/PBdagcon*). Make sure they are in your path variable.

The input files for consensus are:

(1) backbone_raw.fasta by DBG2OLC

(2) DBG2OLC_Consensus_info.txt by DBG2OLC

(3) DBG contigs (in FASTA format)

(4) PacBio reads (in FASTA format)

You can check the N50 of (1) to see if it meets your standard, otherwise keep tuning and don't proceed.

this is to concatenate the contigs and the raw reads for consensus

cat Contigs.txt pb_reads.fasta > ctg_pb.fasta

we need to open a lot of files to distribute the above file into lots of smaller files

ulimit -n unlimited

#run the consensus scripts

sh ./split_and_run_sparc.sh backbone_raw.fasta DBG2OLC_Consensus_info.txt ctg_reads.fasta ./consensus_dir 2 >cns_log.txt

Commands used to assemble other genomes:

The *A. thaliana* Ler-0 dataset:

20x PacBio reads:

./DBG2OLC KmerCovTh 2 AdaptiveTh 0.005 MinOverlap 20 RemoveChimera 1 Contigs Contigs.txt k 17 f ../PacBio/20x.fasta

40x PacBio reads:

./DBG2OLC KmerCovTh 2 AdaptiveTh 0.01 MinOverlap 20 RemoveChimera 1 Contigs Contigs.txt k 17 f ../PacBio/40x.fasta

The *H. sapiens* dataset:

Longest 30x PacBio reads:

./DBG2OLC k 17 KmerCovTh 2 MinOverlap 20 AdaptiveTh 0.01 RemoveChimera 1 Contigs Contigs.txt f 30x.fasta >DBG2OLC_LOG.txt

Illumina-only Assembly

With the rapid advancement of sequencing technology, the length of the accurate NGS reads has also become increasingly longer. As a prototype, we demonstrate that the approach can be extended to existing Illumina data. Existing DBG based assembly algorithms resorted to stretches of perfectly matching *k*-mers and are not robust to sequencing errors. Algorithms required growing *k*-mer sizes to find increasingly perfect overlaps and extensive iterations to exploit the long read information. A costly error correction module was critical in finding these perfect overlaps. In contrast, our work can quickly and reliably find the best read overlaps without per-base level correction. Since our approach of utilizing the long read information is certainly not restricted to low quality ones, we compared the performance of our assembler on longer Illumina reads with several popular assemblers, including SGA [1], SOAPdenovo2 [2], SPAdes3 [3]. Interestingly, for the relatively longer short NGS reads generated from the latest NGS technology, traditional *de Bruijn* graph with a fixed *k*-mer size have already exposed its shortage and produces a non-optimal assembly: smaller *k*-mer fails to resolve repetitive regions, while using a large *k* requires high quality and high coverage data. This pair of contradictory requirements makes it hardly possible to obtain the optimal assembly with limited computational resources. Iterative *de Bruijn* graph may partially deal with the problem at the expense of more computational time, as well as more intricate algorithm design and implementations. For example, an error correction procedure (with an exponential-complexity graph search) would be necessary to produce long and correct *k*-mers. SGA utilized the FM-index [4] to find exact matches, which also poses restriction on the quality of the data. In contrast, our algorithm is robust and poses loose restriction on the quality of sequence reads, and hence can find overlaps efficiently and correctly. A dataset of 50X 150bp Illumina Miseq reads of *E. coli* K-12 MG1655 (Accession no: SRA073308) is used here as a test case. SparseAssembler[5] with k = 31 is called to assemble the initial contigs and it reaches an N50 of 13.5 kbp. The compressed reads were calculated using the contigs and raw Illumina reads. Our overlap graph construction took only around 1 second on this dataset while the compressing is taking most of the computational time, which is roughly two minutes. The total computational time of SparseAssembler and DBG2OLC is 5 minutes. DBG2OLC exhibits excellent adaptability and overall performance compared with leading assemblers for the new types of the NGS data.

Table S4. Assembly performance comparison on the *E.coli* genome using NGS MiSeq reads (Genome size: 4.6M bp)

Assembler	Time (hr)	Memory Peak (GB)	NG50	Identity (%)	NGA50	Misassem-blies	Longest	Sum
SGA	1	2	54,945	100.0%	54,800	0	185,528	4,629,362
SOAPdenovo2	0.1	0.1	54,896	100.0%	52,788	0	160,084	4,600,372
SPAdes3	2	8	112,387	100.0%	107,855	0	327,031	4,659,050
DBG2OLC	0.1	0.1	118,892	100.0%	110,457	0	327,255	4,693,366

Commands for Illumina-only assembly:

The program command is slightly different.

Example command:

./DBG2OLC LD 0 MinOverlap 70 PathCovTh 3 Contigs Contigs.txt k 31 KmerCovTh 0 f ReadsFile1.fa f ReadsFile2.fq f MoreFiles.xxx

There are four critical parameters:

k: k-mer length (max size: 31).

KmerCovTh: # k-mer matches for a contig to be regarded as a genuine anchor, suggest 0-1.

MinOverlap: # 'consistent' k-mers between each pair of reads to be considered to overlap.

PathCovTh: the minimum occurrence for a compressed read for a compressed read to be used, suggest 1-3.

Assembly is reported as DBG2OLC_Consensus.fasta.

The command we used for E. coli Illumina Miseq dataset:

./DBG2OLC k 31 PathCovTh 2 MinLen 50 MinOverlap 31 Contigs Contigs.txt KmerCovTh 0 f Illumina_reads.fasta

4. Dotplots of Alignments to the Reference Genomes

The following commands were used to generate the dot plots:
nucmer -mumreference -b 200 -g 200 -c 200 –p out Reference.fasta Assembly.fasta
mummerplot --png --l --large -f --fat out.delta

Figure S1.The alignment of DBG2OLC assembly of the *S. cer* genome (Y-axis) to the reference created using 454 sequencing (X-axis). 10x PacBio reads and 50x Illumina reads are used.

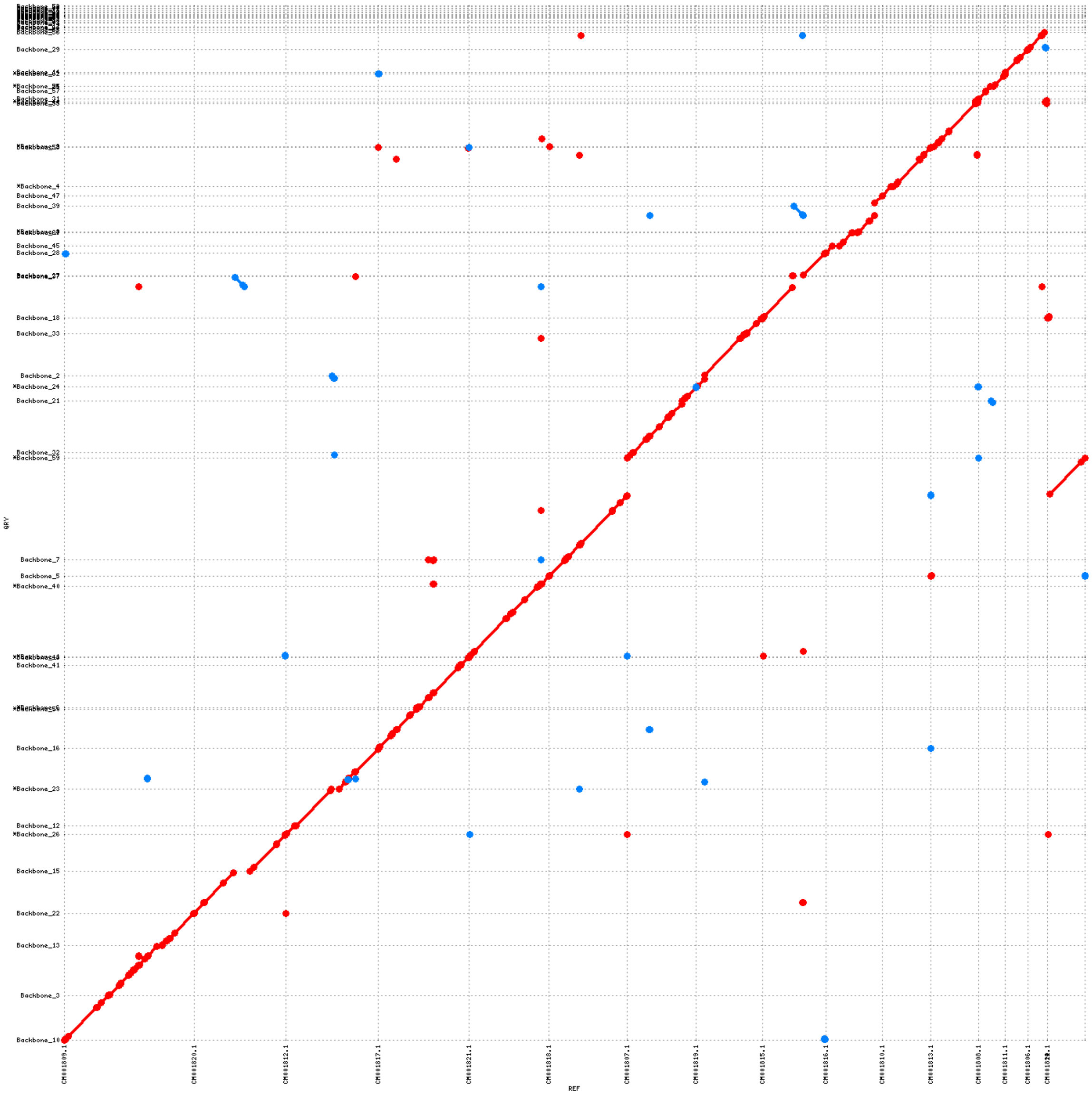

Figure S2.The alignment of DBG2OLC assembly of the *S. cer* genome (Y-axis) to the high coverage PacBio assembly (X-axis). 10x PacBio reads and 50x Illumina reads are used.

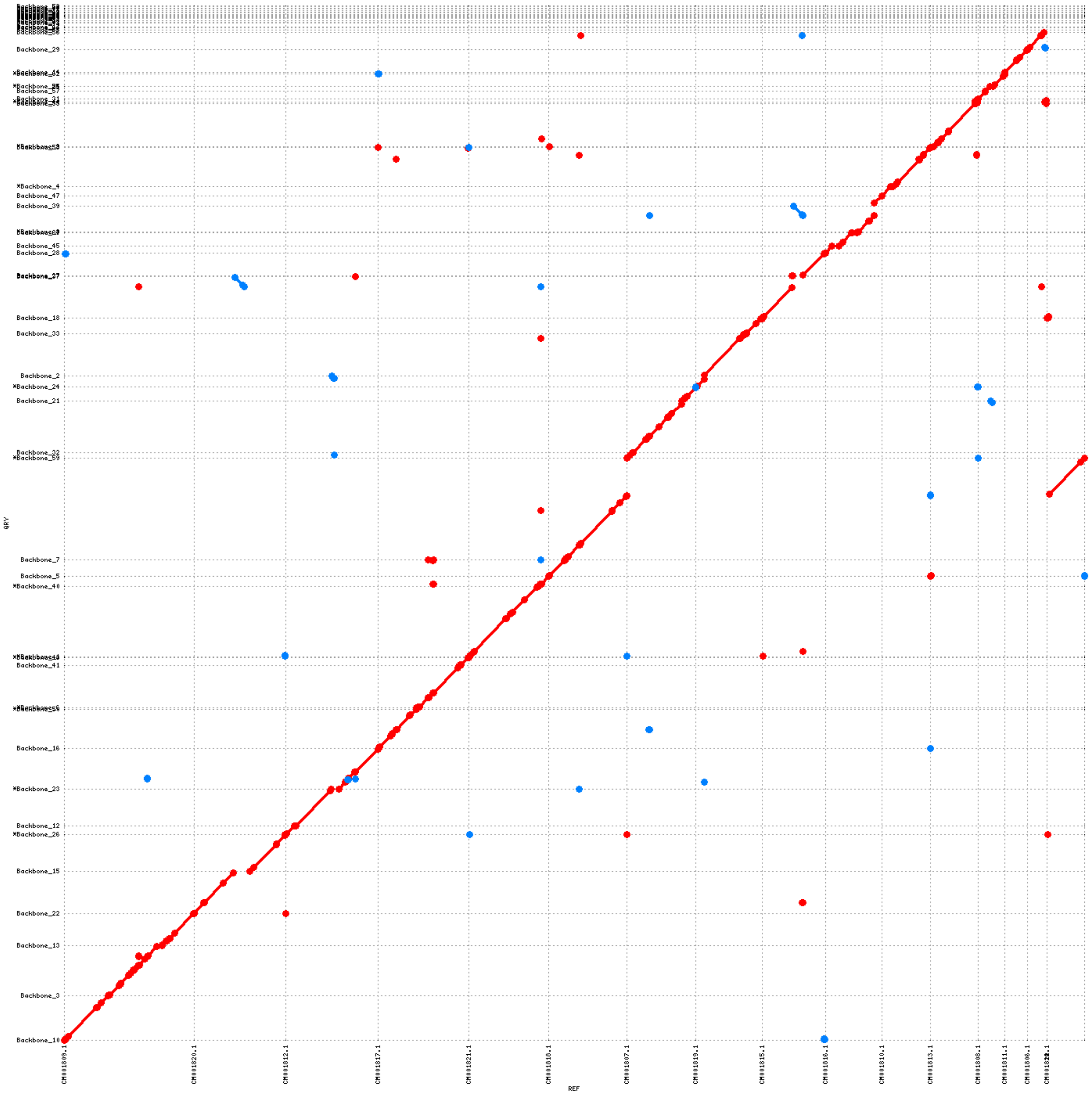

Figure S3.The alignment of DBG2OLC assembly of the *S. cer* genome (Y-axis) to the reference created using 454 sequencing (X-axis). 10x PacBio reads and 50x Illumina reads are used.

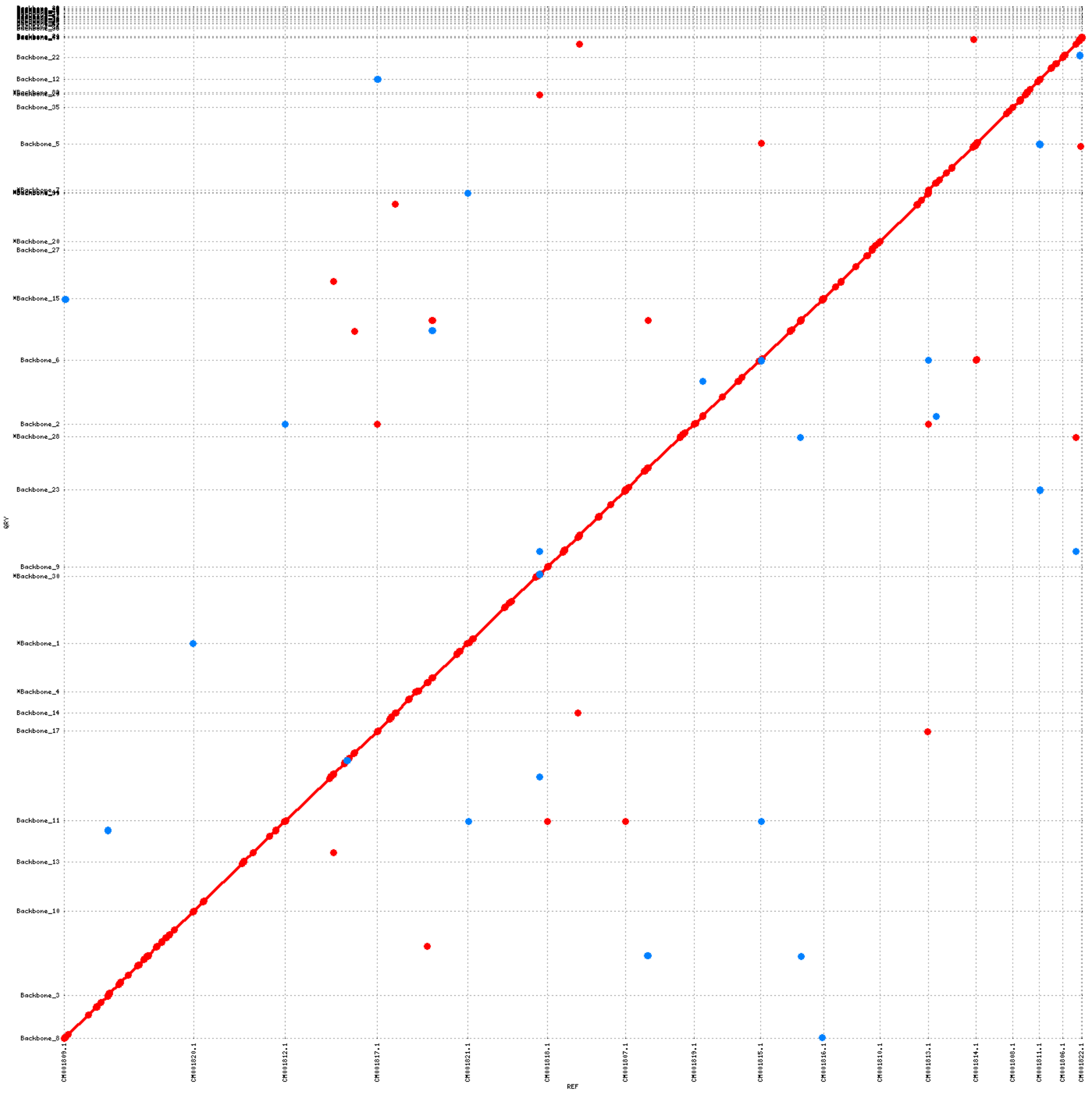

Figure S4.The alignment of DBG2OLC assembly of the *S. cer* genome (Y-axis) to the high coverage PacBio assembly (X-axis). 20x PacBio reads and 50x Illumina reads are used.

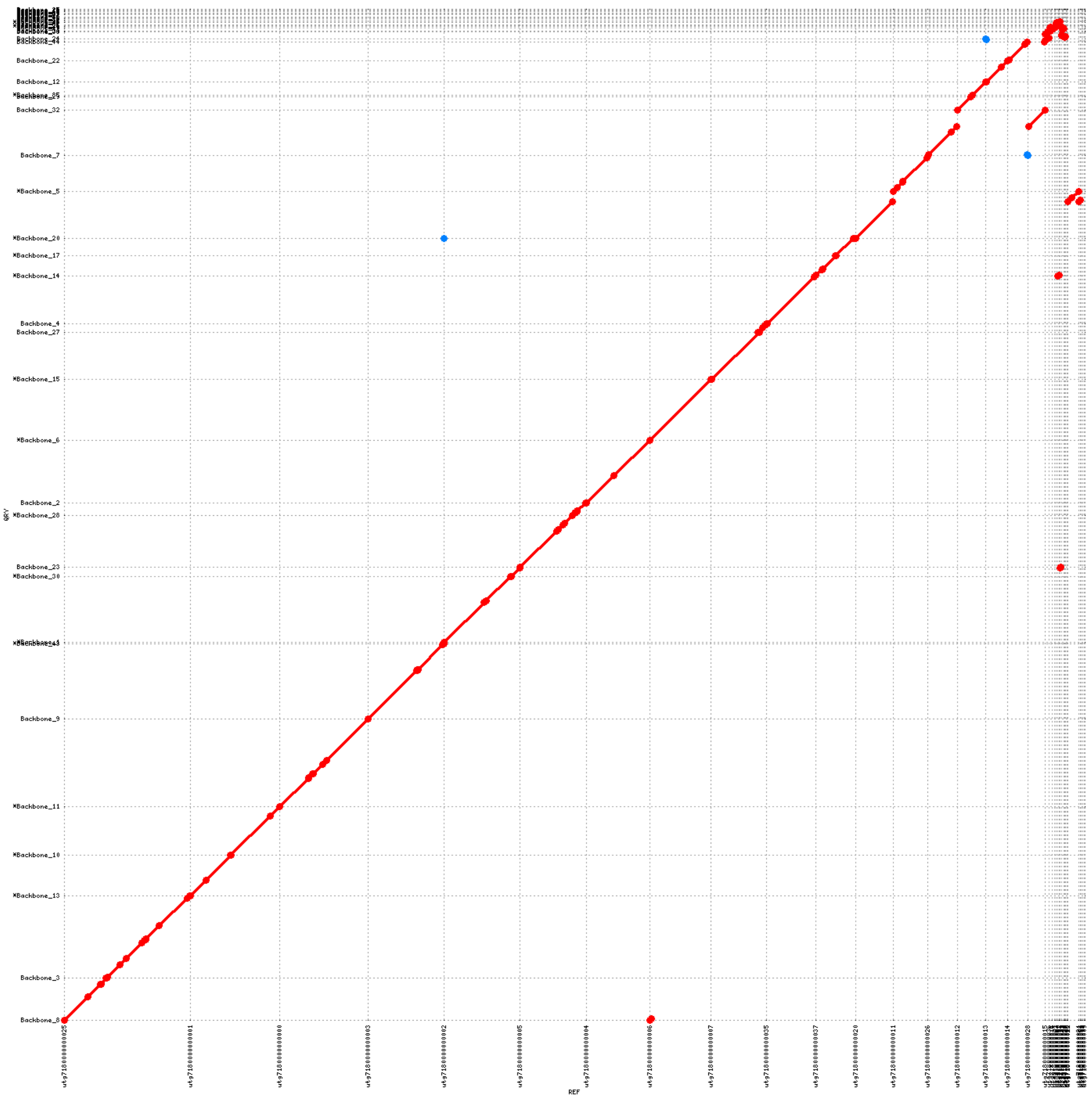

Figure S5.The alignment of DBG2OLC assembly of the *S. cer* genome (Y-axis) to the reference created using 454 sequencing (X-axis). 40x PacBio reads and 50x Illumina reads are used.

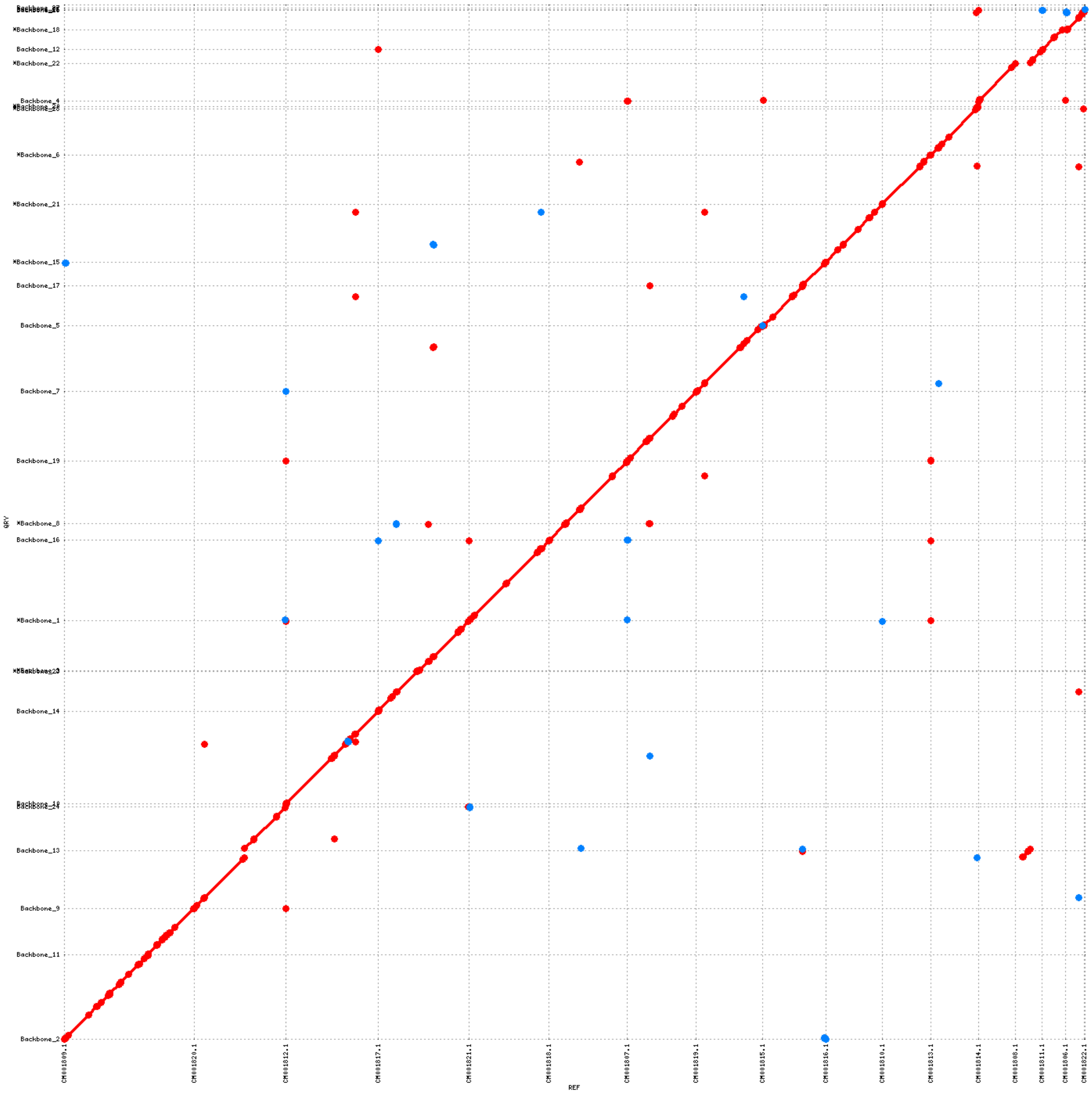

Figure S6.The alignment of DBG2OLC assembly of the *S. cer* genome (Y-axis) to the high coverage PacBio assembly (X-axis). 40x PacBio reads and 50x Illumina reads are used.

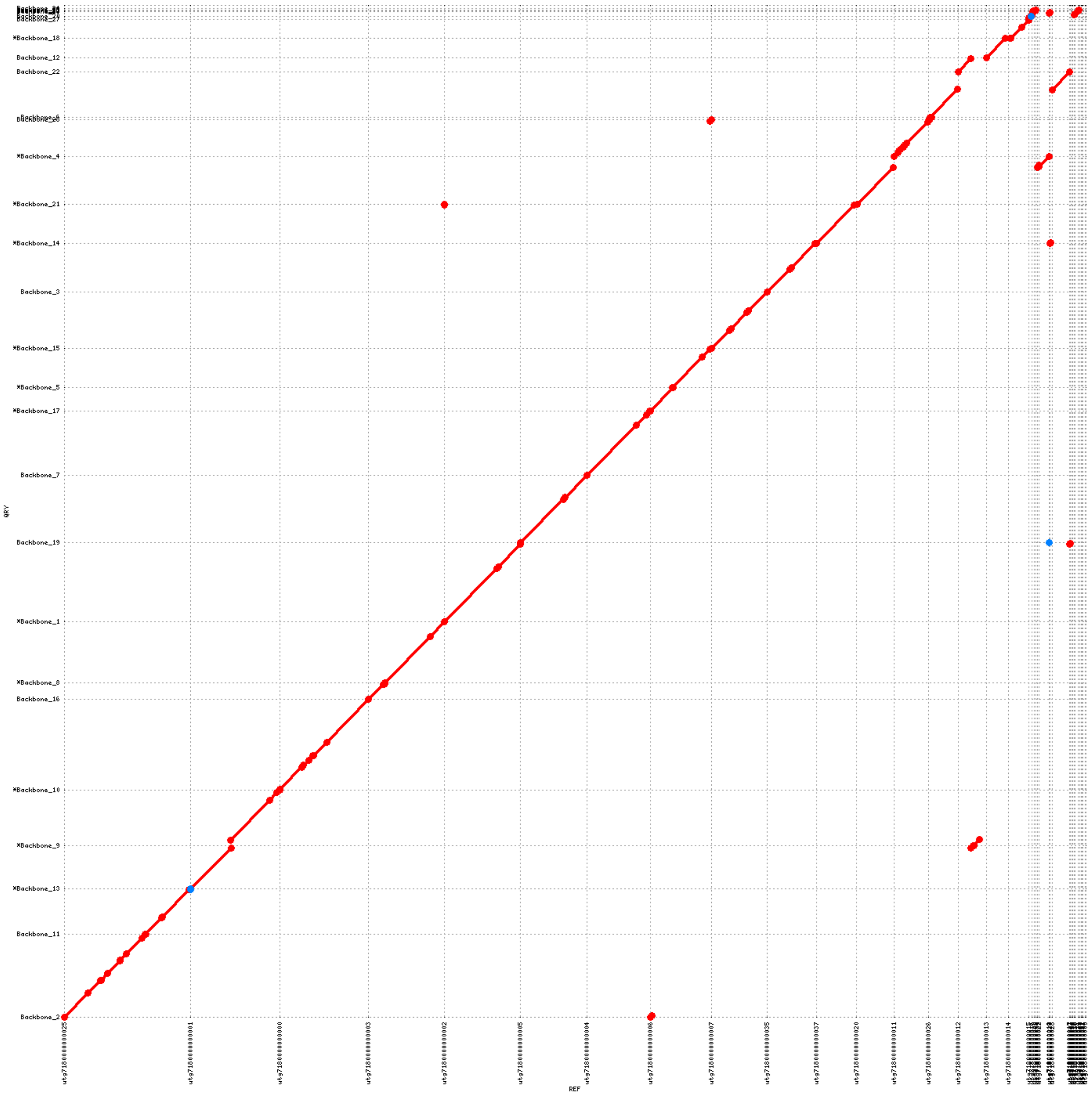

Figure S7.The alignment of DBG2OLC assembly of the *S. cer* genome (Y-axis) to the reference created using 454 sequencing (X-axis). 80x PacBio reads and 50x Illumina reads are used.

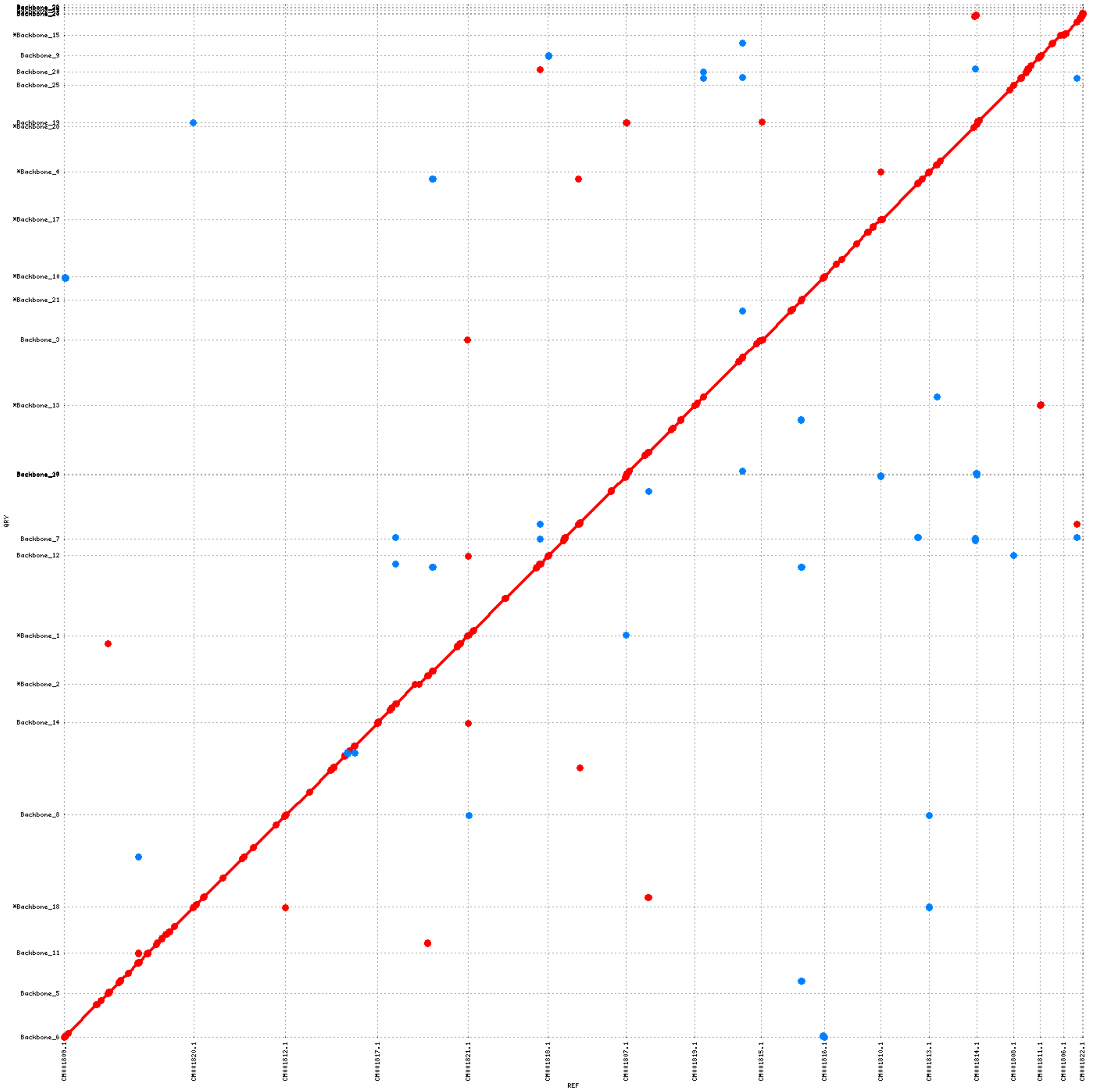

Figure S8.The alignment of DBG2OLC assembly of the *S. cer* genome (Y-axis) to the high coverage PacBio assembly (X-axis). 80x PacBio reads and 50x Illumina reads are used.

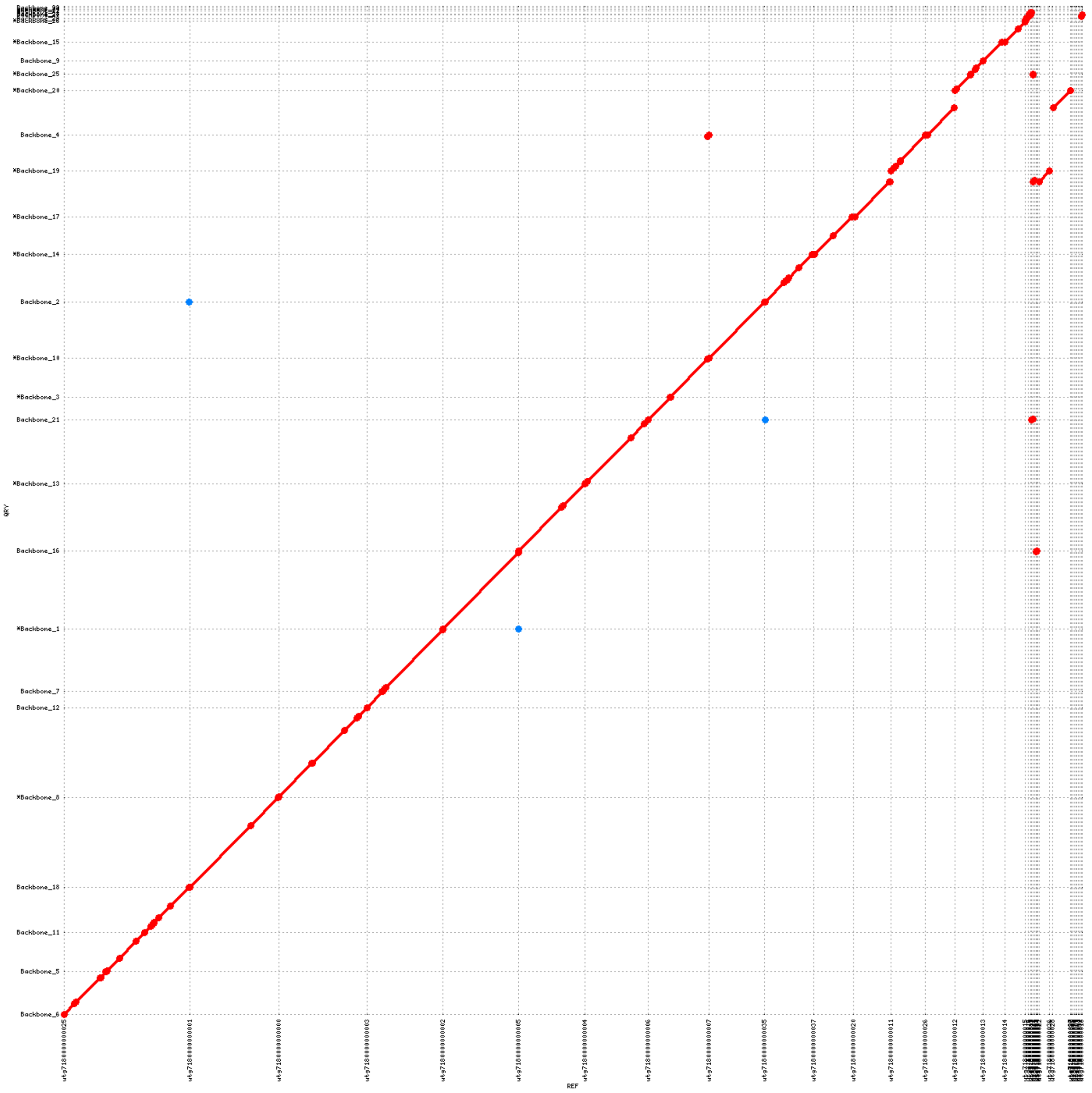

Figure S9. Alignment of high coverage PacBio assembly of *S. cer* genome (Y-axis) to the 454 assembly.

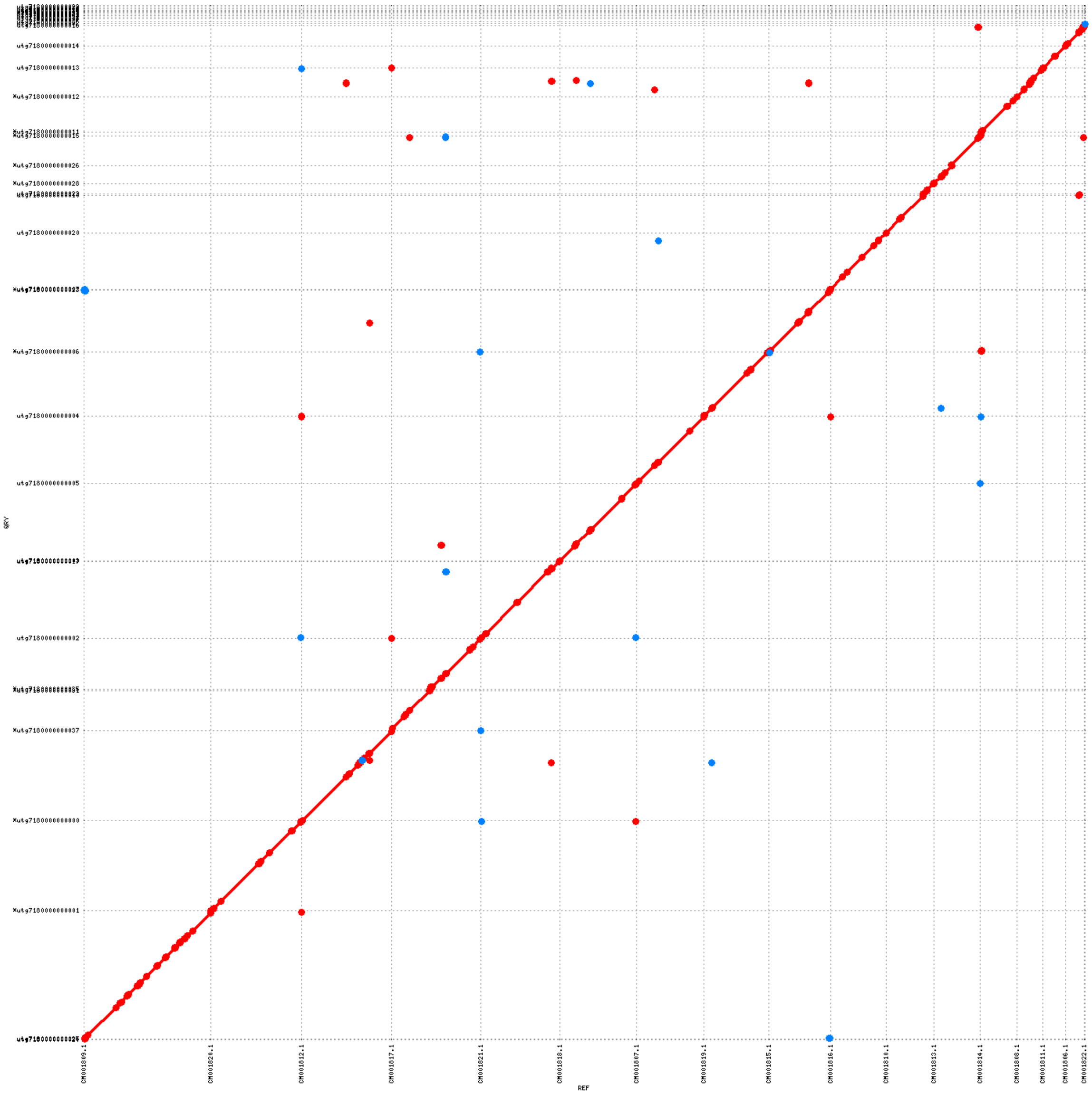

Figure S10.The alignment of DBG2OLC assembly of the *A. thaliana* genome (Y-axis) to the high coverage PacBio assembly (X-axis). 10x PacBio reads and 50x Illumina reads are used.

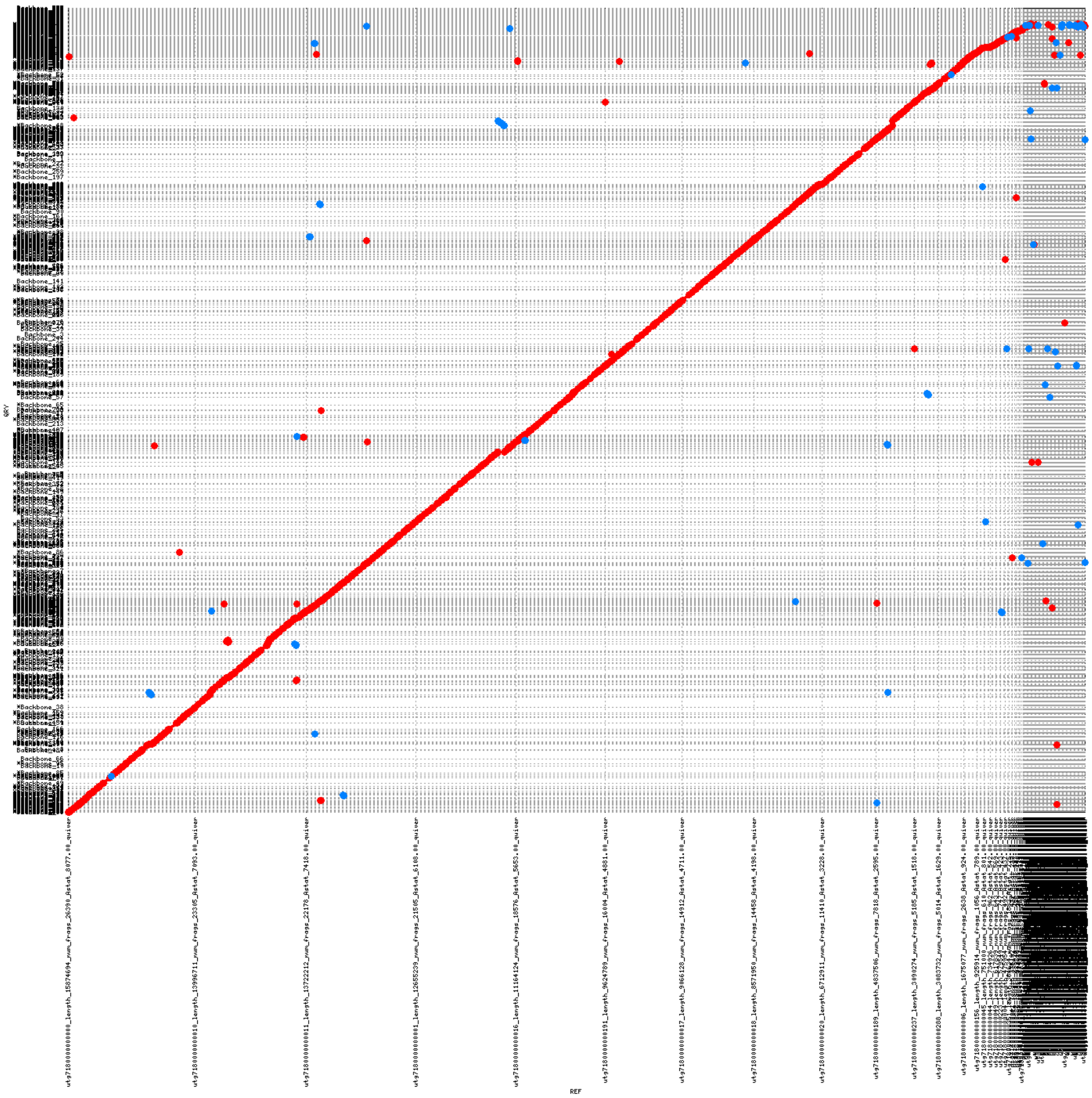

Figure S11.The alignment of DBG2OLC assembly of the *A. thaliana* genome (Y-axis) to the high coverage PacBio assembly (X-axis). 20x PacBio reads and 50x Illumina reads are used.

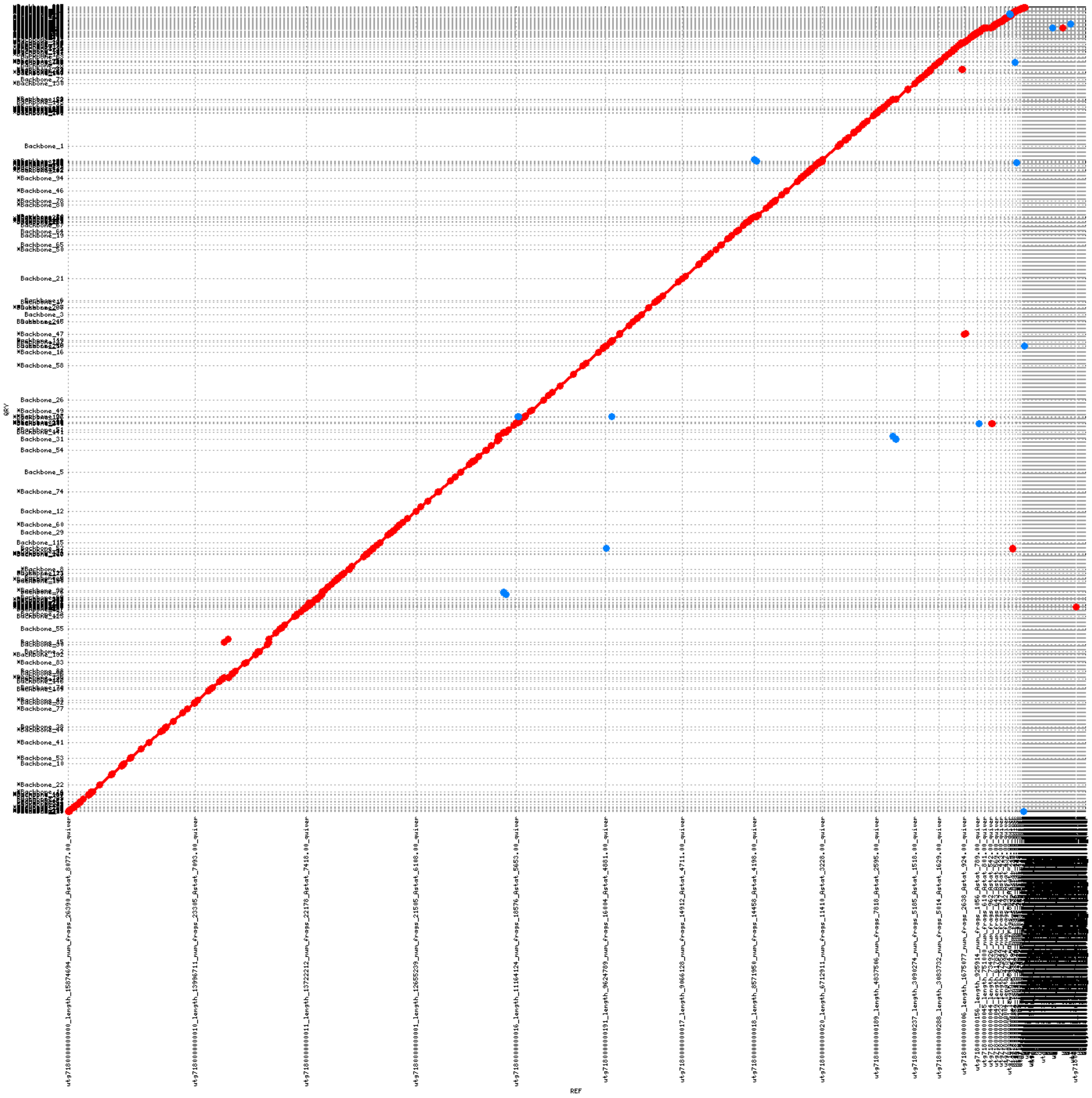

Figure S12.The alignment of DBG2OLC assembly of the *A. thaliana* genome (Y-axis) to the high coverage PacBio assembly (X-axis). 40x PacBio reads and 50x Illumina reads are used.

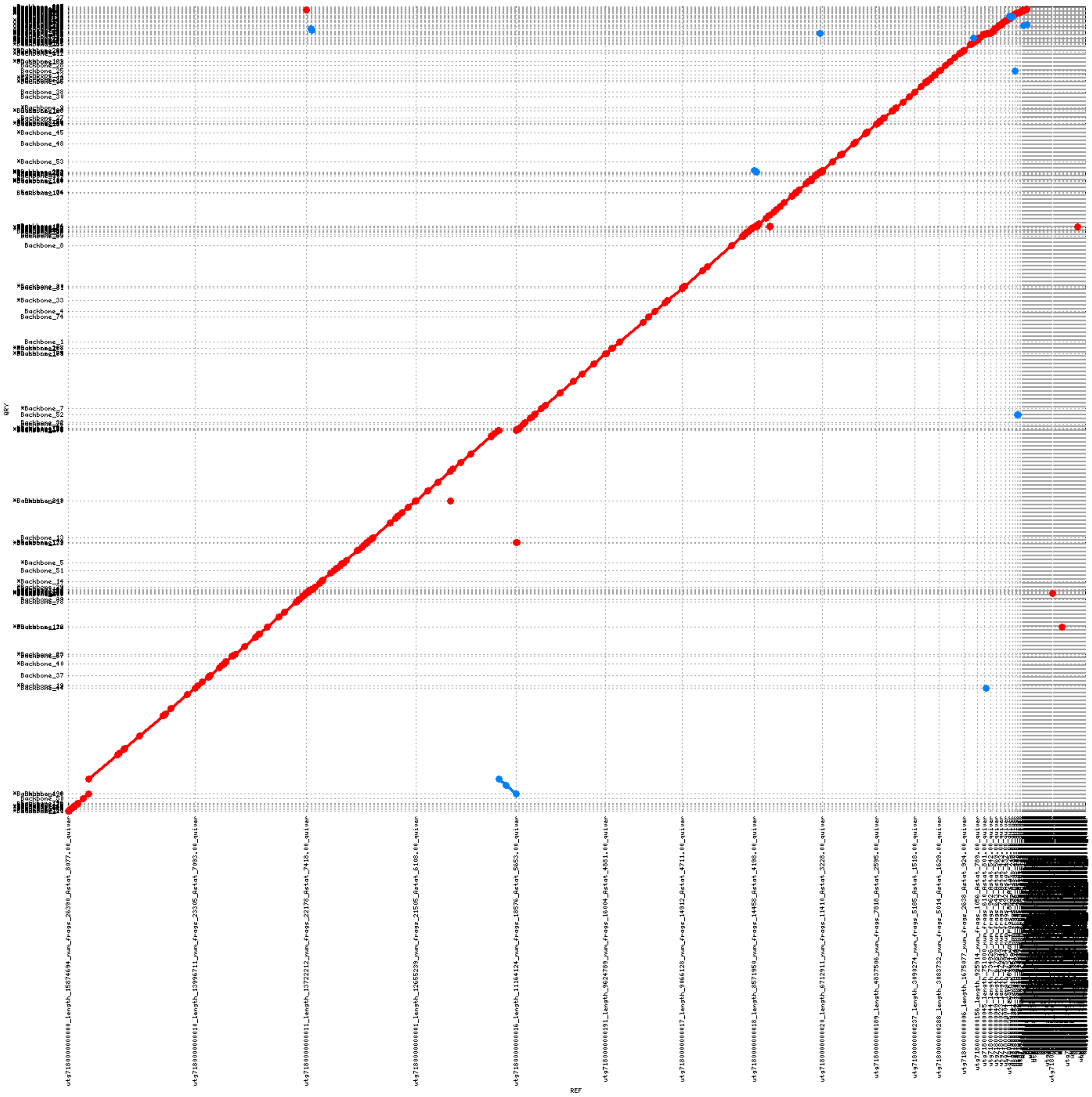

Figure S13.The alignment of DBG2OLC assembly of the *E. coli* genome (Y-axis) to the reference genome (X-axis). 30x Oxford Nanopore reads and 50x Illumina reads are used.

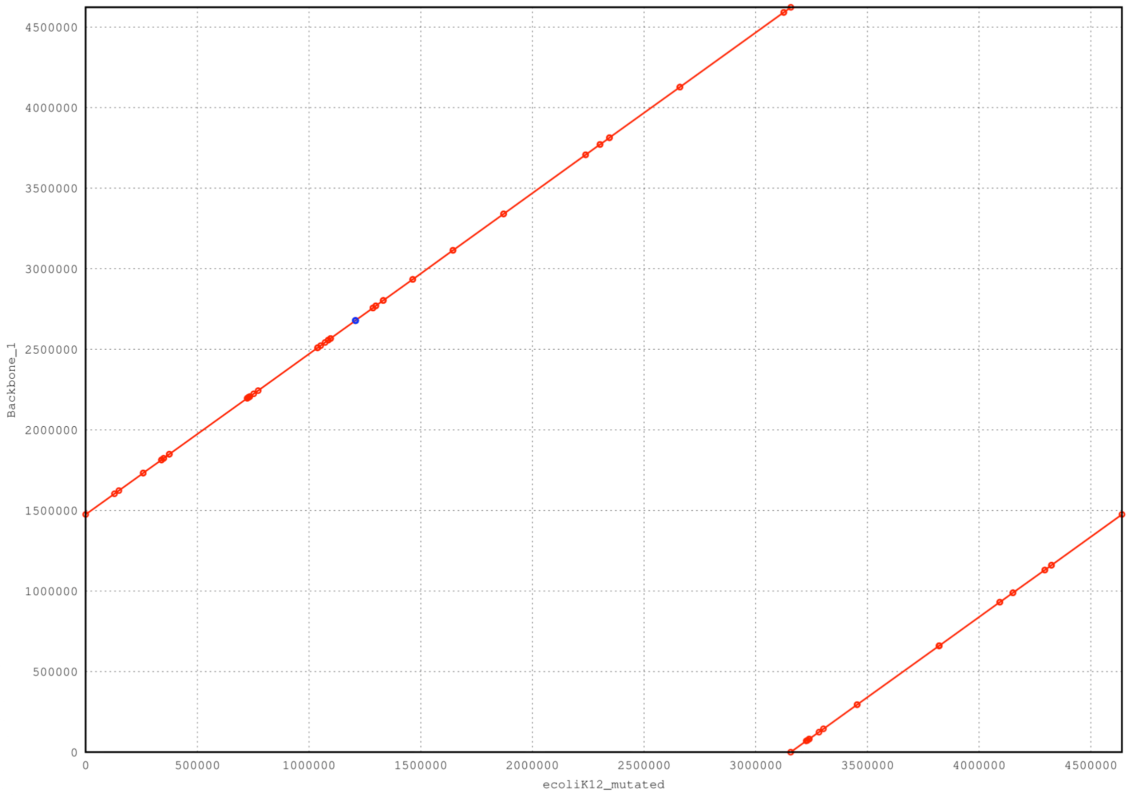

5. Commands used to run other pipelines

MHAP
PBcR -length 500 -partitions 200 -l yeast -s pacbio.spec -fastq 80x.fastq genomeSize=12000000

HGAP
smrtpipe.py -D NPROC=3 -D CLUSTER=BASH -D MAX_THREADS=4 --params=params.xml
xml:input.xml >smrtpipe.log

CA
runCA -p yeast-trim -d yeast-trim -s yeast-trim.spec yeast-untrimmed.frg

PacBioToCA
pacBioToCA -length 500 -partitions 200 -l ec_pacbio -t 16 -s pacbio.spec -fastq 80x.fastq yeast.frg
runCA -p asm -d asm -s asm.spec ec_pacbio.frg

Falcon
fc_run.py fc_run.cfg

ECTools
for i in {0001..000N}; do cd $i; qsub -cwd -j y -t 1:500 ../correct.sh; cd ..; done
runCA -p assembly -d assembly -s assembly.spec organism.cor.frg

Celera assembler specification files can be found in a different attachment.

References

1 Simpson, J. T. & Durbin, R. Efficient de novo assembly of large genomes using compressed data structures. *Genome research* **22**, 549-556, doi:10.1101/gr.126953.111 (2012).

2 Luo, R. *et al.* SOAPdenovo2: an empirically improved memory-efficient short-read de novo assembler. *GigaScience* **1**, 18, doi:10.1186/2047-217X-1-18 (2012).

3 Bankevich, A. *et al.* SPAdes: a new genome assembly algorithm and its applications to single-cell sequencing. *Journal of computational biology : a journal of computational molecular cell biology* **19**, 455-477, doi:10.1089/cmb.2012.0021 (2012).

4 Ferragina, P. & Manzini, G. in *Proceedings. 41st Annual Symposium on Foundations of Computer Science.* 390-398.

5 Ye, C., Ma, Z. S., Cannon, C. H., Pop, M. & Yu, D. W. Exploiting sparseness in de novo genome assembly. *BMC Bioinformatics* **13 Suppl 6**, S1, doi:10.1186/1471-2105-13-S6-S1 (2012).
